# Association Between Self-Efficacy and Mental Health: Perceived Social Support as a Mediator

**DOI:** 10.3390/healthcare14182952

**Published:** 2026-09-10

**Authors:** Aashiq Khan, Irum Zeb, Shuanghu Fang

**Affiliations:** 1 School of Educational Science, Anhui Normal University, Wuhu 241000, China; ashiqkhan.edu@gmail.com; 2 School of Political Science and Public Administration, Henan Normal University, Xinxiang 453007, China; irumzeb.kiu@gmail.com

**Keywords:** self-efficacy, social support, mental health, students, universities

## Abstract

**Highlights:**

**What are the main findings?**

- Self-efficacy was positively associated with mental health among Chinese university students.
- Perceived social support partially mediated the association between self-efficacy and mental health.

**What are the implications of the main findings?**

- Student mental health programs should strengthen both personal confidence and perceived social support.
- Universities should promote supportive peer, family, and institutional environments to improve students’ well-being.

**Abstract:**

**Background:** Mental health is important in higher education because university students often face academic stress and social adjustment challenges. **Objective:** We examined the direct and indirect associations among self-efficacy, perceived social support, and mental health among students at H university in central China. **Methods:** A stratified convenience sample of 581 undergraduate, master’s, and doctoral students completed online or face-to-face self-report questionnaires. Shortened measures were used to reduce respondent burden. Self-efficacy was assessed using six items from the Self-Efficacy Scale, perceived social support using three items from the Multidimensional Scale of Perceived Social Support, and mental health using six items from the 12-item General Health Questionnaire. The measures showed acceptable reliability and validity overall (α = 0.848–0.876; CR = 0.886–0.924; AVE = 0.564–0.801; HTMT < 0.85). SPSS 26 and SmartPLS v4.0 were used to examine the proposed associations with 5000 bootstrap resamples. **Results:** Self-efficacy had a significant positive association with mental health (β = 0.293, *p* < 0.001) and perceived social support (β = 0.721, *p* < 0.001). Perceived social support had a significant positive association with mental health (β = 0.145, *p* = 0.025). The indirect association between self-efficacy and mental health through perceived social support was significant (β = 0.105, *p* = 0.026). The model explained 16.8% of the variance in mental health and 52.0% of the variance in perceived social support. **Conclusions:** In this single-university sample, self-efficacy and perceived social support were positively associated with mental health. The cross-sectional self-report design and convenience sampling prevent causal conclusions and limit generalizability. The findings should also be interpreted cautiously because the shortened measures were not independently validated.

## 1. Introduction

University students worldwide face substantial mental health (MH) challenges, including elevated stress, anxiety, and depressive symptoms, during a developmental period marked by rapid role transitions, academic evaluation, and increasing independence. Mental health problems among university students are also associated with poorer academic functioning [1]. In China, academic stress has been positively associated with anxiety, hopelessness, and depressive symptoms among college students [2]. These conditions make it important to identify psychosocial resources that help students remain well and function effectively during demanding academic and social transitions.

MH is a multidimensional phenomenon with varying emphases in different disciplines. The World Health Organization defines MH as a state of mental well-being that enables individuals to cope with life stress, realize their abilities, learn and work effectively, and contribute to their communities [3]. In the current study, MH is operationalized as general mental health and day-to-day functioning, including concentration, problem-solving, enjoyment of daily life, perceived usefulness, confidence, and sleep disturbance related to worry because university life often entails high workloads, uncertain outcomes, and social comparison; uncertainty about the personal and social resources that support MH is a feasible priority for student development and campus support systems.

One central personal resource is self-efficacy (SE). SE refers to individuals’ beliefs about their capability to organize and execute actions required to attain desired outcomes in specific situations [4]. Social Cognitive Theory (SCT) considers SE as a major determinant of motivation and behavior, affecting whether someone sets challenging goals and whether they persist in the face of obstacles and recover from setbacks [4]. SCT further proposes that SE is shaped through four principal sources: mastery experiences, vicarious learning, verbal persuasion, and the regulation and interpretation of emotional and physiological states [4]. For university students, these sources are of high concern: recurrent academic successes and failures, exposure to the performance of peers, feedback about success or failure from instructors or family, and stress responses throughout the day can all be sources of efficacy perceptions. As SE increases, students are more likely to appraise demanding situations as manageable, use adaptive coping strategies, and remain engaged even when outcomes are uncertain. Consistent with this reasoning, previous research has indicated that higher SE is related to better MH and lower psychological distress among university students, in part because SE is related to more effective coping and perseverance under stress [5,6].

Although SE is frequently studied as a predictor of MH, university students do not cope with stressors solely through personal beliefs and strategies. Their social environments also influence the manner in which they experience and respond to academic and life demands. Perceived social support (PSS) reflects an individual’s belief that emotional and instrumental support is available and sufficient when they need it, especially from significant sources such as the family, friends and significant others [7]. Importantly, PSS reflects perceived availability and sufficiency of support rather than the objective amount of help received, and perceptions may influence coping even when actual support use varies. PSS has been associated with MH among university students, with greater social support generally associated with better well-being and mental health [8]. Among students, perception of the availability of support may serve to render academic challenges less threatening and make it easier to seek help and reach effective problem-solutions, as well as buffer emotional distress in the event of multiple stressors.

The Chinese university context provides a relevant setting for examining these relationships because academic stress has been associated with anxiety, hopelessness, and depressive symptoms among Chinese college students [2]. Perceived social support has also been associated with adaptive coping among university students in mainland China [9]. However, the present study does not assume that these relationships are unique to Chinese students or necessarily differ from those observed elsewhere. Instead, it examines whether the proposed associations are evident among students at one Chinese university, a context that remains comparatively underrepresented in the literature.

Previous research has associated higher SE with better MH and lower psychological distress among students [10,11]. PSS has similarly been associated with better MH and lower anxiety and depressive symptoms. However, studies have assigned different roles to PSS. Lu and Wang [12] examined PSS as a moderator of the association between SE and psychological well-being, whereas Khan and Zeb [13] identified PSS as a mediator between psychological capital and MH. Fu [14] also examined PSS as a mediator linking social connectedness, optimism, and adaptive coping. Therefore, mediation by PSS is not presented as entirely novel. The more specific contribution of this study is to examine the direct and indirect associations among SE, PSS, and MH within one model and among students at one Chinese university. SCT and COR theory provide complementary explanations for these associations. SCT explains how efficacy beliefs may be associated with coping, persistence, and engagement when individuals encounter challenging situations [4,15]. COR theory conceptualizes SE and PSS as personal and social resources that may coexist and support psychological adjustment under stress [16]. Thus, SCT addresses students’ efficacy-related engagement with their environment, while COR theory explains how personal and social resources may operate together in relation to MH. Their integration provides a focused rationale for examining the indirect association rather than proposing an entirely new theoretical mechanism.

Because the study is cross-sectional, the proposed paths represent theoretically informed statistical associations and do not establish temporal ordering or causality. The alternative possibility that PSS precedes SE also remains plausible and requires longitudinal examination. Accordingly, the present study examines whether SE is directly associated with MH and indirectly associated with MH through PSS. The following hypotheses are proposed:

**H1.**  *SE is positively associated with MH.*

**H2a.**  *SE is positively associated with PSS.*

**H2b.**  *PSS is positively associated with MH.*

**H2c.**  *SE has a significant positive indirect association with MH through PSS.*

## 2. Materials and Methods

Data were collected using a cross-sectional research design from H University, located in central China. A stratified convenience sampling approach was used to recruit university students, and data were gathered through both face-to-face and online questionnaires. Strata were defined by gender, age group, and qualification level to obtain responses from different student groups. Recruitment targets were set across these strata, and participants were invited through available university networks, including classroom-based face-to-face distribution and online WeChat distribution. The recruitment frame consisted of currently enrolled undergraduate, master’s, and doctoral students at H University who were accessible through these classroom-based and online university networks. Recruitment within the defined strata remained convenience-based rather than probability-based. Of the 581 valid responses, 380 questionnaires were collected online via WeChat, and the remaining 201 were collected face-to-face. Participants provided written informed consent before participation. Participants were eligible as currently enrolled university students. No additional exclusion criteria were applied. Questionnaires with substantial missing data or invalid response patterns were excluded from analysis. A priori power analysis was conducted using G*Power (version 3.1) for linear multiple regression (fixed model, R^2^ deviation from zero) to determine the required sample size. The analysis specified a medium effect size (f^2^ = 0.15), α = 0.05, power (1 − β) = 0.95, and 3 predictors, indicating a minimum required sample size of 138. To account for potential nonresponse and invalid questionnaires [17], we inflated the target sample size and distributed 600 questionnaires, yielding 581 valid responses (96.83%). As a sensitivity check using the achieved sample size (N = 581; α = 0.05; 3 predictors), the study had 95% power to detect effects as small as f^2^ ≈ 0.03, indicating that the final sample exceeded the a priori requirement and did not compromise the targeted effect size. Sample characteristics are presented in Table 1. Of the 581 participants, 370 were female (63.68%), and 211 were male (36.32%). Most participants were aged 21–25 years (*n* = 300, 51.64%), followed by 18–20 years (*n* = 157, 27.02%) and 26–30 years (*n* = 124, 21.34%). Regarding majors, 250 students were in the humanities (43.00%), 221 in science (38.00%), and 110 in engineering (19.00%). With respect to study level, 258 participants were undergraduates (44.40%), 235 were master’s students (40.45%), and 88 were PhD students (15.15%). These groups were combined because the primary analysis examined overall associations among enrolled university students. However, study level was not examined as a moderator, and the pooled analysis should not be interpreted as demonstrating equivalent relationships across the three groups.

### 2.1. Adaptation of Measurement Instruments

To reduce respondent burden and improve clarity for the student sample, we used shortened versions of established instruments by retaining items that best matched the study’s operational definitions. Following common scale-adaptation practice, item retention emphasized conceptual relevance to the university context [18]. Item selection was finalized before data collection based on conceptual alignment, relevance to university students, and respondent burden. The study-specific measures were evaluated using factor loadings, Cronbach’s alpha, rho_A, composite reliability, AVE, and HTMT. They should be interpreted as adapted measures, and comparisons with findings obtained using the complete instruments should be made cautiously.

### 2.2. Self-Efficacy

Self-efficacy was measured using six items adapted from the 10-item General Self-Efficacy Scale (GSE) developed by Schwarzer and Jerusalem [19]. The scale assesses individuals’ general confidence in coping with difficult or unexpected situations and managing challenging demands. The retained items assessed students’ confidence in dealing with unexpected events, maintaining motivation during difficulties, solving problems effectively, handling unexpected situations, overcoming challenges through effort and persistence, and completing difficult tasks. The example items included “I am confident I could deal efficiently with unexpected events”; “Even when facing difficulties, I can stay motivated to succeed”; “I am confident in my ability to solve problems effectively”; and “I can handle unexpected situations well”. Responses were recorded on a 4-point Likert scale ranging from 1 = Disagree to 4 = Strongly agree, with higher scores indicating stronger self-efficacy. The six-item scale demonstrated acceptable internal consistency in the present study (Cronbach’s α = 0.862).

### 2.3. Mental Health

Mental health was measured using 6 items adapted from the 12-item General Health Questionnaire (GHQ-12) developed by Goldberg [20]. The GHQ-12 is widely used to screen general mental health and psychological distress, and we shortened it to reduce respondent burden while retaining items suitable for assessing students’ psychological distress and everyday functioning. The six retained items assessed concentration, problem-solving difficulty, enjoyment of daily life, feeling useful, loss of confidence, and insomnia due to excessive worry. Items included “To what extent can you concentrate when doing something?” and “How often do you experience insomnia due to excessive worry?” Responses were recorded using the original four-point Likert response format. The positively oriented items were scored from 1 to 4, whereas the negatively oriented items assessing difficulty solving problems (MH2), loss of confidence (MH5), and insomnia due to excessive worry (MH6) were reverse-coded so that all six items had the same direction. Higher scores therefore indicated better general mental health and lower psychological distress. The revised mental health scale showed acceptable reliability (α = 0.848).

### 2.4. Perceived Social Support

Perceived social support was measured using items adapted from the Multidimensional Scale of Perceived Social Support (MSPSS) developed by Zimet and Dahlem [7]. The original MSPSS is a 12-item instrument with three subdimensions: family, friends, and significant others. In the present study, three items were retained to assess students’ overall perceived availability of social support across important interpersonal sources. The retained items were: “When I face difficulties, some people will be there for me,” “My friends, family, and others support me when I face hard times,” and “I can get emotional help and support from my family when I need it.” These three indicators were modeled as reflective indicators of a single latent construct, Perceived Social Support, in the structural equation model. They reflected students’ general appraisal that support was available from people around them, including family, friends, and other important social ties. Responses were recorded on a 7-point Likert scale ranging from 1 = strongly disagree to 7 = strongly agree. In this study, the adapted perceived social support scale demonstrated acceptable reliability (α = 0.876), Table 2.

### 2.5. Data Analysis

This study employed IBM SPSS Statistics (version 26) and SmartPLS (version 4.0) for data analysis. After reverse-coding the negatively worded mental health items, mean scores were calculated across the retained items for each construct to obtain descriptive statistics. To maintain consistency between the correlation matrix and the structural model, the correlations reported in Table 3 are latent variable correlations obtained from the same PLS-SEM model used to estimate the structural paths. For PLS-SEM, the individual items were entered directly as reflective indicators; therefore, items were not summed or averaged before measurement- and structural-model estimation. Thus, the participant-level construct mean used for descriptive reporting was not entered as an indicator in the PLS-SEM model; the correctly oriented item-level variables were used instead. Before model estimation, data normality was assessed using univariate skewness, excess kurtosis, and Mardia’s multivariate skewness and kurtosis statistics [21].

Although CB-SEM was feasible given the sample size and model structure, PLS-SEM was selected because the data significantly violated multivariate normality and the analysis focused on explaining variance in PSS and MH. Statistical significance and confidence intervals were assessed through nonparametric bootstrapping with 5000 resamples [22,23]. SEM was used as a theory-consistent analytic framework for examining associations among SE, PSS, and MH, rather than for establishing temporal ordering or causal relations. The measurement model was tested using PLS-SEM to assess indicator loading, reliability, convergent validity (AVE ≥ 0.50), and discriminant validity (HTMT < 0.85–0.90) [23]. After confirming measurement quality (Cronbach’s alpha and composite reliability ≥ 0.70), the structural model was evaluated for direct and indirect effects, including collinearity (VIF < 3.3), explained variance (R2), and model fit (SRMR < 0.08) [24]. Preliminary subgroup analyses were also conducted to examine whether SE, PSS, and MH differed according to participant characteristics. Gender comparisons were examined using independent-samples tests, whereas age group, major, and study level were examined using one-way analyses of variance. Effect sizes were reported using Hedges’ g for two-group comparisons and η^2^ for comparisons involving more than two groups. These analyses were treated as exploratory and were not part of the hypothesized structural model. Demographic variables were not subsequently included as covariates because the structural model was specified a priori to test the focal theoretical associations. Their exclusion was based on the theoretical scope of the model rather than statistical significance alone, and possible residual confounding is acknowledged.

## 3. Results

This section presents the main findings of the study. It includes descriptive statistics, reliability and validity results, model fit, direct relationships, mediation results, and hypothesis testing.

### 3.1. Descriptive Statistics

Descriptive statistics were examined to summarize participants’ scores on the study variables. For the complete sample (N = 581), the mean scores were 3.72 (SD = 0.64) for self-efficacy, 3.73 (SD = 0.80) for perceived social support, and 3.09 (SD = 0.49) for mental health. Subgroup sample sizes and percentages are reported in Table 1. Because inferential subgroup comparisons were outside the hypothesized model, no claims regarding gender or educational-level differences are made. The latent variable correlations obtained from the same PLS-SEM model showed that self-efficacy was positively correlated with perceived social support (r = 0.721) and mental health (r = 0.398). Perceived social support was also positively correlated with mental health (r = 0.357) presented in Table 3.

### 3.2. Preliminary Subgroup Analyses

Table 4 presents preliminary analyses examining whether SE, PSS, and MH differed across participant characteristics. No significant gender differences were observed for SE (t = −0.09, *p* = 0.929, Hedges’ g = −0.008), PSS (t = 0.62, *p* = 0.535, g = 0.052), or MH (t = 1.62, *p* = 0.105, g = 0.139). Similarly, no significant differences across age groups were found for SE (F = 0.09, *p* = 0.910, η^2^ < 0.001), PSS (F = 0.19, *p* = 0.824, η^2^ = 0.001), or MH (F = 2.51, *p* = 0.082, η^2^ = 0.009). No significant differences were observed across study levels for SE (F = 0.205, *p* = 0.815, η^2^ = 0.001), PSS (F = 0.722, *p* = 0.486, η^2^ = 0.002), or MH (F = 0.242, *p* = 0.785, η^2^ = 0.001). Thus, undergraduate, master’s, and doctoral students showed broadly comparable scores on the three study constructs. Academic-discipline differences were nonsignificant for SE (F = 1.99, *p* = 0.138, η^2^ = 0.007) and PSS (F = 2.48, *p* = 0.085, η^2^ = 0.008). A statistically significant but small difference was observed for MH across academic disciplines (F = 4.41, *p* = 0.013, η^2^ = 0.015). Post-hoc comparisons indicated that the difference was primarily between humanities and engineering students, whereas the remaining pairwise comparisons were not statistically significant. Given the small effect size and exploratory nature of these analyses, this finding should be interpreted cautiously.

### 3.3. Measurement Model

This study tested the direct effects of Self-Efficacy (SE) on Mental Health (MH) and Perceived Social Support (PSS), as well as the indirect effect of SE on MH via PSS. Before estimating these relationships, we assessed the reliability and validity of the measurement model. All item loadings were approximately 0.69 or higher, indicating adequate indicator reliability [24]. Internal consistency was acceptable. Cronbach’s alpha and composite reliability values exceeded 0.70 for all constructs. Cronbach’s alpha ranged from 0.848 to 0.876, rho_A ranged from 0.865 to 0.879, and composite reliability ranged from 0.886 to 0.924. Convergent validity was established because AVE values for all constructs exceeded 0.50. Detailed factor loadings, reliability indices, and AVE values are reported in Table 5.

Discriminant validity was further assessed using the heterotrait–monotrait ratio (HTMT). As shown in Table 6, the HTMT values were 0.827 between self-efficacy and perceived social support, 0.443 between self-efficacy and mental health, and 0.393 between perceived social support and mental health. The highest value was 0.827, which remained below the conservative threshold of 0.85. These findings support the discriminant validity of the three constructs.

### 3.4. Multicollinearity and Model Fit

Collinearity was assessed using variance inflation factors (VIFs). The inner-model VIF was 1.000 for SE → PSS and 2.083 for both SE → MH and PSS → MH, indicating no problematic multicollinearity. The SRMR value was 0.073, below the recommended threshold of 0.08, as shown in Table 7. SRMR was treated as supplementary model-fit information, while the structural model was primarily evaluated using path coefficients, confidence intervals, R^2^, f^2^, and predictive assessment.

### 3.5. Structural Equation Model

This study employed PLS-SEM to examine the direct and indirect statistical associations among the study variables. Bootstrapping with 5000 resamples and 95% confidence intervals was applied to assess the significance of the direct and indirect associations. All results reported in the correlation, measurement, and structural-model analyses were obtained from the same final model specification.

The findings support Hypothesis H1, indicating that SE was significantly positively associated with MH (β = 0.293, t = 4.633, *p* < 0.001, SE = 0.063, 95% CI [0.171, 0.416]). Similarly, supporting Hypothesis H2a, SE was significantly positively associated with PSS (β = 0.721, t = 29.164, *p* < 0.001, SE = 0.025, 95% CI [0.670, 0.769]). Finally, supporting Hypothesis H2b, PSS was significantly positively associated with MH (β = 0.145, t = 2.242, *p* = 0.025, SE = 0.065, 95% CI [0.017, 0.272]).

The mediation analysis showed a significant indirect association between self-efficacy and mental health through perceived social support. This finding is statistically consistent with partial mediation but does not establish a causal or temporal process. The indirect association was significant (β = 0.105, t = 2.228, *p* = 0.026, SE = 0.047, 95% CI [0.012, 0.197]), while the direct association between self-efficacy and mental health remained significant (β = 0.293, *p* < 0.001), thereby supporting Hypothesis H2c. The total association was 0.398, and the variance accounted for was 26.3% (VAF = 0.105/0.398 × 100). Thus, approximately 26.3% of the total statistical association between SE and MH was represented by the indirect component through PSS as presented in Table 8.

The explanatory power of the model was assessed using the coefficient of determination (R^2^). The R^2^ values were 0.168 for MH (adjusted R^2^ = 0.166) and 0.520 for PSS (adjusted R^2^ = 0.519). Specifically, SE and PSS jointly explained 16.8% of the variance in MH, whereas SE explained 52.0% of the variance in PSS.

The local effect sizes were assessed using f^2^. SE had an f^2^ effect size of 0.050 on MH, PSS had an f^2^ effect size of 0.012 on MH, and SE had an f^2^ effect size of 1.083 on PSS. Thus, the largest local effect was observed for the association between SE and PSS, whereas the individual effects of SE and PSS on MH were considerably smaller. These results show that statistical significance should be interpreted together with practical magnitude [25,26]. For the single-predictor PSS equation, the effect size was calculated as f^2^ = R^2^/(1 − R^2^).

PLSpredict was used to assess out-of-sample predictive relevance. The Q^2^ predict values were positive for MH (Q^2^ predict = 0.150; RMSE = 0.927; MAE = 0.727) and PSS (Q^2^predict = 0.517; RMSE = 0.698; MAE = 0.507), as shown in Table 9. These positive values indicate that the model had predictive relevance relative to the indicator-mean benchmark. Because linear-model benchmark values were unavailable, the relative degree of predictive performance was not classified.

**Figure 1:**
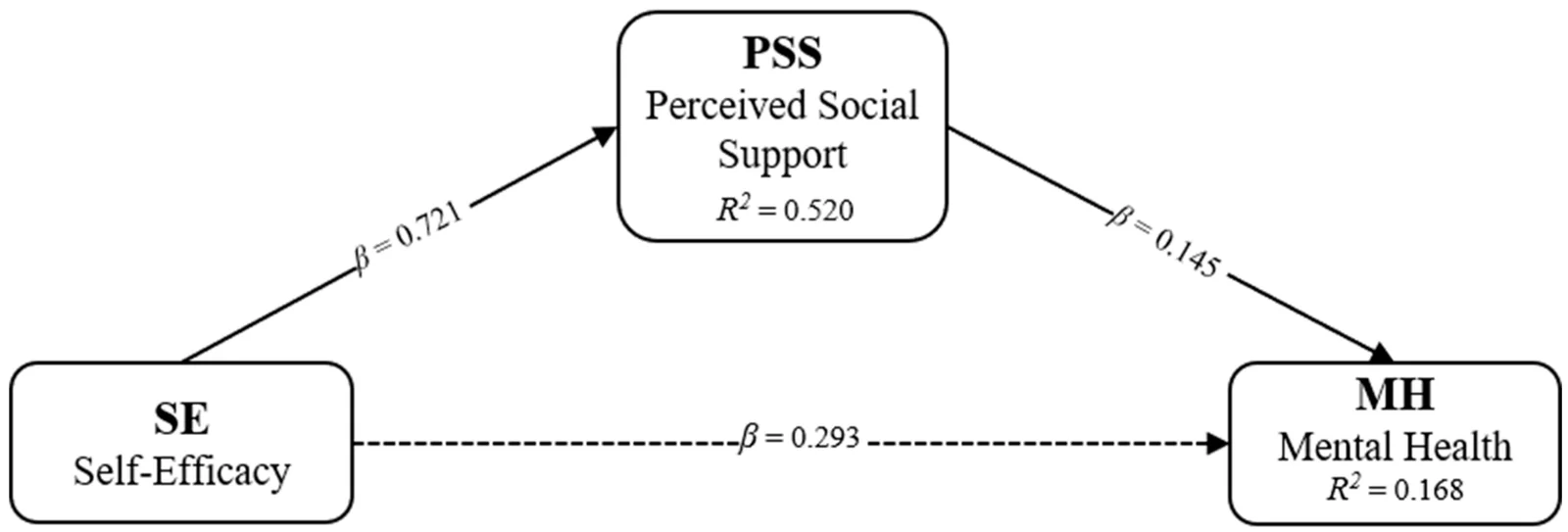
Structural model with standardized path coefficients for the relationships among self-efficacy (SE), perceived social support (PSS), and mental health (MH).

Given the cross-sectional design, all reported direct, indirect, and total associations are interpreted as statistical relationships consistent with the proposed theoretical framework rather than evidence of temporal ordering or causal processes. Figure 1 presents the direct and indirect associations in the proposed model.

## 4. Discussion

This study identified positive associations among self-efficacy, perceived social support, and mental health among students at one Chinese university. It also identified a significant indirect association between self-efficacy and mental health through perceived social support. These findings are interpreted as cross-sectional statistical relationships rather than evidence of causal or temporal processes.

The positive association between self-efficacy and mental health is consistent with previous evidence linking stronger efficacy beliefs with lower psychological distress and better student adjustment [5,6,25]. SCT provides one interpretation of this relationship by proposing that efficacy beliefs are associated with how individuals appraise challenges, maintain effort, and respond to difficulties [4]. Students reporting stronger self-efficacy may also perceive academic demands as more manageable and report greater confidence in their coping abilities. However, the present data cannot determine whether stronger self-efficacy supports mental health, whether better mental health strengthens efficacy beliefs, or whether both are influenced by other personal and contextual factors.

SCT and COR theory make distinct but complementary contributions to interpreting the indirect association. SCT explains why efficacy beliefs may be associated with greater confidence in coping with challenges and persistence when difficulties arise [4,15]. COR theory explains why personal resources, such as self-efficacy, and social resources, such as perceived support, may coexist and jointly relate to psychological adjustment under stress [16]. Thus, SCT focuses on general efficacy beliefs relevant to coping and persistence, whereas COR theory focuses on the clustering and accumulation of personal and social resources. Combining the theories provides a fuller interpretation of the observed associations, although the cross-sectional design does not directly test the behavioral or resource-accumulation processes proposed by either theory.

The indirect association through PSS should not be interpreted as an entirely novel mediation mechanism. Previous studies have assigned different roles to social support. Lu and Wang [12] examined PSS as a moderator of the association between self-efficacy and psychological well-being, whereas Khan and Zeb [13] identified PSS as a mediator between psychological capital and mental health. Fu [14] also examined PSS as a mediator linking social connectedness, optimism, and adaptive coping among university students in mainland China. The contribution of the present study is therefore more specific: it examines the direct and indirect associations among self-efficacy, PSS, and mental health within one model and among students at one Chinese university.

The comparatively high explanatory power for PSS requires careful interpretation. Self-efficacy explained 52.0% of the variance in PSS, indicating that efficacy beliefs had a comparatively strong statistical association with students’ perceptions of available support. However, this cross-sectional association should not be interpreted as evidence that self-efficacy determines students’ perceptions of social support. PSS may also be associated with the availability and quality of family and peer relationships, personality traits, help-seeking preferences, socioeconomic circumstances, academic workload, institutional climate, and access to university services. The remaining variance may also reflect characteristics of the shortened three-item PSS measure. Longitudinal research is required to determine the temporal relationship between SE and PSS.

Similarly, the model explained 16.8% of the variance in mental health, leaving 83.2% unexplained. Mental health may also be associated with academic stress, personality, socioeconomic status, financial difficulties, physical health, family relationships, coping strategies, previous psychological difficulties, and access to professional support. In addition, the alternative ordering PSS → SE → MH remains theoretically plausible because supportive relationships may strengthen efficacy beliefs through encouragement, feedback, and social persuasion [4]. Longitudinal and cross-lagged studies are required to compare these alternative pathways and establish their temporal ordering.

Overall, the findings provide context-specific evidence that personal and perceived social resources are jointly associated with student mental health. They extend previous research by applying the complementary perspectives of SCT and COR theory to an indirect-association model in a Chinese university setting. However, the results should not be generalized to all Chinese university students, and the observed associations should be interpreted cautiously because of the cross-sectional design, convenience sampling, self-report measures, and study-specific shortened instruments.

### 4.1. Practical Implications

Because the study was cross-sectional, the following implications are tentative and require evaluation through longitudinal or controlled intervention research. The participating university and institutions with similar contexts could pilot programs that jointly address students’ confidence in managing academic challenges and their awareness of available social support. Such programs could combine coping and problem-solving workshops with peer mentoring, faculty–student mentorship, and clear referral pathways to student-support services [4,26]. Their effectiveness should be assessed using pre-intervention and post-intervention measures and appropriate comparison groups.

University mental health practitioners could assess both students’ efficacy beliefs and their perceived access to family, peer, faculty, and institutional support during counselling. Support-network mapping and structured referral procedures could help identify students who report limited available support. However, whether these practices improve mental health must be established through direct evaluation rather than inferred from the present associations.

Teachers could make academic support more visible through structured peer collaboration, supportive feedback, and clear information about available assistance. These practices may be especially relevant for students who report low confidence or limited perceived support, although their effectiveness should be evaluated in the local educational context.

University administrators and institutional policymakers could support accessible counselling, peer-support, and mentoring systems and require these initiatives to include outcome evaluation. Decisions to expand such programs should be based on longitudinal or experimental evidence rather than the present cross-sectional findings alone.

### 4.2. Limitations

This study has four main limitations. First, the cross-sectional design does not establish causality or temporal ordering. The SE–PSS–MH pathway represents a statistical indirect association, and the alternative direction of PSS → SE → MH remains plausible. Future longitudinal, cross-lagged, and experimental studies should compare these pathways.

Second, all constructs were measured using self-report and study-specific shortened instruments, which may introduce common method and response biases. The shortened measures were not independently validated through expert assessment, pilot testing, comparison with the complete scales, or a separate validation sample. In addition, the self-efficacy measure was an adapted six-item subset of the GSE and therefore assessed broad coping efficacy rather than domain-specific academic or social self-efficacy. Consequently, the present findings cannot determine whether domain-specific efficacy beliefs would show different associations with PSS or mental health. Future studies should use complete validated scales or independently validate the shortened forms and consider domain-specific efficacy measures when testing specific academic or interpersonal mechanisms.

Third, the stratified convenience sample was drawn from one Chinese university and included more female than male participants. Undergraduate, master’s, and doctoral students were combined in the primary structural model; however, preliminary analyses showed no significant differences in SE, PSS, or MH across study levels. In addition, online and face-to-face administration may have created different response conditions. The nonprobability recruitment process may also have introduced self-selection and response bias. Therefore, the findings should be interpreted within the context of the participating university rather than generalized to all Chinese university students. These factors limit generalizability and may conceal subgroup or administration-mode differences. Future studies should include multiple universities, more balanced samples, consistent administration procedures, and measurement-invariance or multigroup analyses.

Finally, the model explained 16.8% of the variance in mental health, leaving 83.2% unexplained. Other personal, academic, and contextual factors including academic stress, anxiety, financial difficulties, family relationships, institutional support, coping, resilience, and psychological capital may also be relevant. Therefore, the model should be interpreted as a partial rather than comprehensive explanation of students’ mental health.

## 5. Conclusions

This study examines the associations of self-efficacy and perceived social support with students’ mental health. The findings suggest that self-efficacy is positively associated with students’ mental health. Furthermore, perceived social support showed a significant indirect association between self-efficacy and mental health. However, because study-specific shortened measures were used, these findings should be interpreted cautiously and replicated using complete or independently validated instruments.

## Figures and Tables

**Table 1:** Sample characteristics (N = 581).

Category	No. of Respondents	Percentage
Gender		
Male	211	36.32
Female	370	63.68
Age (years)		
18–20	157	27.02
21–25	300	51.64
26–30	124	21.34
Major		
Humanities	250	43.00
Science	221	38.00
Engineering	110	19.00
Study level		
Undergraduate	258	44.40
Masters	235	40.45
PhD	88	15.15

**Table 2:** Content of analyzed indicators.

Construct	Indicator	Item Content
Self-Efficacy	SE1	I am confident I could deal efficiently with unexpected events.
	SE2	Even when facing difficulties, I can stay motivated to succeed.
	SE3	I am confident in my ability to solve problems effectively.
	SE4	I can handle unexpected situations well.
	SE5	I believe I can overcome challenges through hard work and persistence.
	SE6	I feel capable of accomplishing tasks, even when they are difficult.
Mental health	MH1	To what extent can you concentrate when doing something?
	MH2	How frequently do you feel unable to solve problems?
	MH3	How often do you enjoy your daily life?
	MH4	How often do you feel like a useful person?
	MH5	How often have you lost your confidence?
	MH6	How often do you experience insomnia due to excessive worry?
Perceived social support	PSS1	When I face difficulties, some people will be there for me.
	PSS2	My friends, family, and others support me when I face hard times.
	PSS3	I can get emotional help and support from my family when I need it.

**Table 3:** Descriptive statistics and correlations among the study variables (N = 581).

Constructs	Mean	SD	1	2	3
1. Self-Efficacy	3.72	0.64	-		
2. Perceived Social Support	3.73	0.8	0.721	-	
3. Mental Health	3.09	0.49	0.398	0.357	-

Note. Correlations are latent variable correlations obtained from the PLS-SEM model.

**Table 4:** Preliminary subgroup comparisons.

Constructs	Outcome	Group Descriptive, M (SD)	*t*	*p*	Effect Size
Gender	SE	Male 3.72 (0.66); Female 3.72 (0.63)	t = −0.09	0.929	g = −0.008
	PSS	Male 3.75 (0.74); Female 3.71 (0.83)	t = 0.62	0.535	g = 0.052
	MH	Male 3.13 (0.48); Female 3.06 (0.49)	t = 1.62	0.105	g = 0.139
Age Group	SE	18–20: 3.71 (0.66); 21–25: 3.72 (0.64); 26–30: 3.74 (0.63)	F = 0.09	0.91	η^2^ < 0.001
	PSS	18–20: 3.75 (0.73); 21–25: 3.71 (0.83); 26–30: 3.74 (0.80)	F = 0.19	0.824	η^2^ = 0.001
	MH	18–20: 3.16 (0.45); 21–25: 3.05 (0.51); 26–30: 3.09 (0.47)	F = 2.51	0.082	η^2^ = 0.009
Academic Major	SE	Humanities 3.75 (0.66); Engineering 3.61 (0.65); Sciences 3.75 (0.60)	F = 1.99	0.138	η^2^ = 0.007
	PSS	Humanities 3.78 (0.76); Engineering 3.58 (0.89); Sciences 3.75 (0.79)	F = 2.48	0.085	η^2^ = 0.008
	MH	Humanities 3.15 (0.47); Engineering 2.98 (0.53); Sciences 3.07 (0.48)	F = 4.41	0.013	η^2^ = 0.015
Qualification Level	SE	Undergraduate: 3.73 (0.62); Master’s: 3.72 (0.67); PhD: 3.68 (0.60)	F = 0.205	0.815	η^2^ = 0.001
	PSS	Undergraduate: 3.77 (0.77); Master’s: 3.70 (0.85); PhD: 3.68 (0.72)	F = 0.722	0.486	η^2^ = 0.002
	MH	Undergraduate: 3.07 (0.52); Master’s: 3.10 (0.46); PhD: 3.08 (0.45)	F = 0.242	0.785	η^2^ = 0.001

**Table 5:** Reliability and validity.

Scales	Factor Loading	(α)	Rho_a	CR	AVE
Self-Efficacy		0.862	0.867	0.897	0.594
SE1	0.695				
SE2	0.759				
SE3	0.735				
SE4	0.821				
SE5	0.777				
SE6	0.830				
Mental Health		0.848	0.865	0.886	0.564
MH1	0.692				
MH2	0.766				
MH3	0.774				
MH4	0.757				
MH5	0.747				
MH6	0.769				
Perceived Social Support		0.876	0.879	0.924	0.801
PSS1	0.876				
PSS2	0.925				
PSS3	0.883				

**Table 6:** Heterotrait–monotrait ratio (HTMT).

Constructs	1	2	3
1. Self-Efficacy	-		
2. Perceived Social Support	0.827	-	
3. Mental Health	0.443	0.393	-

**Table 7:** Collinearity and Model Fit.

Structural Relationship/Fit Index	Estimates
SE → PSS, VIF	1
SE → MH, VIF	2.083
PSS → MH, VIF	2.083
SRMR	0.073

**Table 8:** Direct and indirect associations.

Paths	β	Mean	SE	CI	t	*p*	Results
SE → MH	0.293	0.295	0.063	[0.171, 0.416]	4.633	<0.001	Significant
SE → PSS	0.721	0.722	0.025	[0.670, 0.769]	29.164	<0.001	Significant
PSS → MH	0.145	0.147	0.065	[0.017, 0.272]	2.242	0.025	Significant
SE → PSS → MH	0.105	0.106	0.047	[0.012, 0.197]	2.228	0.026	Significant
R^2^ MH = 0.168; adjusted R^2^ MH = 0.166. R^2^ PSS = 0.520; adjusted R^2^ PSS = 0.519. Total association = 0.398; VAF = 26.3%.							

Note. β = standardized path coefficient; SE = standard error; CI = 95% confidence interval.

**Table 9:** Predictive relevance.

Construct	Q^2^predict	RMSE	MAE
MH	0.150	0.927	0.727
PSS	0.517	0.698	0.507

## Data Availability

The data supporting the findings of this study are available from the first author upon reasonable request, subject to ethical and privacy considerations.

## Abbreviations

The following abbreviations are used in this manuscript:

SE	Self-Efficacy
MH	Mental Health
PSS	Perceived Social Support
SCT	Social Cognitive Theory
COR	Conservation of Resources Theory
PLS-SEM	Partial Least Squares Structural Equation Modeling
SPSS	Statistical Package for the Social Sciences
SmartPLS	Smart Partial Least Squares
GHQ	General Health Questionnaire
MSPSS	Multidimensional Scale of Perceived Social Support
AVE	Average Variance Extracted
CR	Composite Reliability
VIF	Variance Inflation Factor
SRMR	Standardized Root Mean Square Residual
CI	Confidence Interval
